# Genome Sequences of Six Prophages Isolated from Staphylococcus pseudintermedius Strains Recovered from Human and Animal Clinical Specimens

**DOI:** 10.1128/MRA.00387-19

**Published:** 2019-07-11

**Authors:** Juliette R. K. Wipf, Douglas R. Deutsch, Lars F. Westblade, Vincent A. Fischetti

**Affiliations:** a Laboratory of Bacterial Pathogenesis and Immunology, The Rockefeller University, New York, New York, USA; b Department of Pathology and Laboratory Medicine, Weill Cornell Medicine, New York, New York, USA; c Division of Infectious Diseases, Department of Medicine, Weill Cornell Medicine, New York, New York, USA; Loyola University Chicago

## Abstract

Staphylococcus pseudintermedius is a common bacterial pathogen in companion animal medicine and has demonstrated zoonotic potential. Here, we report six new Staphylococcus pseudintermedius prophage genomes of the *Siphoviridae* family, identified in isolates recovered from human and canine clinical specimens.

## ANNOUNCEMENT

Staphylococcus pseudintermedius is a frequently isolated opportunistic pathogen of dogs and other animals, mainly causing pyoderma, wound infections, and otitis media (1–3); it also causes infections in humans, mainly affecting skin and soft tissues, with canines as a presumed source of infection (4–10). Methicillin-resistant Staphylococcus pseudintermedius infections have emerged in both companion animal and human medicine over the last decade and have emphasized the clinical importance of this opportunistic pathogen (3–5, 8, 10, 11).

The mobile elements involved in antibiotic resistance development and host adaptation of S. pseudintermedius are under investigation (3, 12–16). In Staphylococcus aureus, intra- and extrachromosomally located prophages play a significant role in virulence and adaptation processes (17–20). The impact of prophages on other staphylococcal species is expected to occur to a comparable extent (14, 17, 21). In S. pseudintermedius, it has recently been shown that potent leukocidin toxins are encoded on a degenerate prophage (22). Here, we report six novel prophage genomes of S. pseudintermedius, isolated from two human and two canine clinical strains (Table 1). Strains were isolated using standard of clinical care bacteriology cultures (23). Genomic DNA (gDNA) was extracted from strains grown overnight at 37°C in Bacto brain heart infusion (Becton, Dickinson, and Company, Sparks, MD, USA) using the Genomic-tip 100/G with 1.5 μg/ml lysostaphin added to buffer B1 (Qiagen, Hilden, Germany). To detect any plasmidial or episomal prophages, the extrachromosomal DNA (exDNA) of the strains was isolated as previously described (18, 20). Libraries of gDNA and exDNA were prepared using the TruSeq DNA library preparation kit version 2 (Illumina, Inc., San Diego, CA, USA). Sequencing was performed using a high-output kit on the Illumina NextSeq 500 platform, creating 1 × 75-bp reads. Reads were quality controlled with the ShortRead Bioconductor package version 1.28.0 (http://bioconductor.org/packages/ShortRead/) and *de novo* assembled using the Geneious assembler on Geneious version 10.0 set to medium to low sensitivity (Biomatters, Auckland, New Zealand) (24). The PHAge Search Tool Enhanced Release (PHASTER) Web service was used to identify contigs containing prophage sequences (25). The genomes of phiSP15-1, phiSP44-1, and phiSP119-2 were found to be complete on single contigs. For the remaining prophages, preliminary PHASTER Web service alignment was used to assign contigs to prophage structural regions and create prophage genome scaffolds (25). The phiSP119-1 genome was distributed over two contigs, while the phiSP38-1 and phiSP119-3 genomes were distributed over three contigs. Scaffolds were closed by PCR and Sanger sequencing (Genewiz, South Plainfield, NJ, USA). phiSP38-1 contained a 1,783-bp gap between contigs located in the prophage DNA replication region, which was closed by primer walking (Genewiz). Open reading frames (ORFs) of closed prophage genomes were identified using the Rapid Annotations using Subsystems Technology (RAST) v. 2.0 server service (26). Hypothetical functions of the predicted ORFs were subsequently determined by alignment to protein sequences and conserved domains in the BLASTp program (http://blast.ncbi.nlm.nih.gov/Blast.cgi) and the Swiss Institute of Bioinformatics Prosite database (http://prosite.expasy.org/), with priority given to Prosite predictions. Over 150 hypothetical proteins were identified in the novel prophages.

**TABLE 1:** Data for the six prophages isolated from four Staphylococcus pseudintermedius strains

Parameter	Data for prophage:					
	phiSP15-1	phiSP38-1	phiSP44-1	phiSP119-1	phiSP119-2	phiSP119-3
Strain	14-29-15	14-29-38	14-29-44	14-29-119	14-29-119	14-29-119
Isolation source	Human blood	Canine surgical implant	Canine skin	Human sinus	Human sinus	Human sinus
MLST^*a*^	1045^*e*^	84	892	568	568	568
*mecA*^*b*^	Negative	Positive	Negative	Positive	Positive	Positive
No. of reads for gDNA/exDNA	8,523,888/6,783,702	8,019,433/5,077,526	5,031,963/6,912,836	9,385,394/5,906,351	9,385,394/5,906,351	9,385,394/5,906,351
No. of contigs of ≥1,000 bp for gDNA/exDNA	56/52	53/58	59/53	65/101	65/101	65/101
*N*_50_ for gDNA/exDNA (bp)	94,254/87,646	93,334/98,185	90,846/102,595	89,522/105,092	89,522/105,092	89,522/105,092
GenBank accession no.	MK075001	MK075002	MK075003	MK075004	MK075005	MK075006
Coverage^*c*^ for gDNA/exDNA (×)	254/194	305/196	181/510	344/37	273/20	471/38
Prophage size (bp)	43,756	40,765	39,156	44,497	40,011	41,416
Phage type	Sfi11-like	Sfi21-like	Sfi11-like	Sfi21-like	Sfi21-like	Sfi11-like
GC content (%)	35.4	36.0	35.8	36.6	34.1	35.6
Complete prophage genome^*f*^	Yes	Yes	Yes	Yes	No^*g*^	Yes
Putative *attL/attR*^*d*^	CTTGCTCTCCGTATTTT	GTCCCTAATGGGTCCCTAAAAATT	TGATACCGTTTT	GCCTGCAATAGGTGGGGT	Unknown^*g*^	GGGTCCCTAAAAATT

a MLST, multilocus sequence type. Sequence types were determined as described previously (29). Genomic DNA was obtained by lysostaphin lysis (30).

b *mecA*, gene encoding penicillin binding protein 2a. The *mecA* status of the isolates was determined by Wu and colleagues (23).

c Coverage of prophages in gDNA and exDNA sequencing was determined by the Bowtie 2 assembler version 2.3.2 on medium to low sensitivity on Geneious version 11.1.5 (Biomatters, Auckland, New Zealand) (31).

d *attL/attR*, chromosomal integration site or left and right flanking direct repeat. Direct repeats were identified using the Geneious Repeat Finder plugin on Geneious version 10.0 (https://www.geneious.com/plugins/repeat-finder/).

e Sequence type 1045 is a never-before-sequenced type of S. pseudintermedius (https://pubmlst.org/spseudintermedius/).

f The prophage contains all the structural modules of a *Siphoviridae* required for entering both the lytic and lysogenic states (17).

g Integrase and *attL/attR* not identified. phiSP119-2 is a possible remnant or incomplete phage.

In S. aureus, it has been shown that sequencing of exDNA can identify plasmidial or episomal prophages that would otherwise remain undetected (18, 20). The prophages of this study were identified in both the genomic as well as the extrachromosomal compartment and therefore may exist as episomal (“active”) prophages (20). In contrast to S. aureus, where prophages harboring virulence factors are commonly associated with clinical strains, no known virulence factors were detected in the identified S. pseudintermedius prophages (27, 28). Further experimental approaches are necessary to determine the functions of the hypothetical proteins identified in these novel prophages.

### Data availability.

The prophage sequences of this study have been deposited in NCBI GenBank under the accession numbers MK075001, MK075002, MK075003, MK075004, MK075005, and MK075006 (Table 1). The raw reads are available under the NCBI Sequence Read Archive accession numbers SRR8957054, SRR8957055, SRR8957056, SRR8957057, SRR8957058, SRR8957059, SRR8957060, and SRR8957061.
